# FEM-Based Design and Micromachining of a Ratchet Click Mechanism in Mechanical Watch Movements

**DOI:** 10.3390/mi16080875

**Published:** 2025-07-29

**Authors:** Alessandro Metelli, Giuseppe Soardi, Andrea Abeni, Aldo Attanasio

**Affiliations:** Department of Mechanical and Industrial Engineering, University of Brescia, Via Branze 38, 25123 Brescia, Italy; alessandro.metelli@unibs.it (A.M.); giuseppe.soardi@unibs.it (G.S.); andrea.abeni@unibs.it (A.A.)

**Keywords:** micromachining, anti-reverse mechanism, FEM modeling

## Abstract

The ratchet click mechanism in mechanical watch movements is a micro-component essential to prevent the unwinding of the caliber mainspring, providing secure energy storage during recharging. Despite its geometrical simplicity, the ratchet click undergoes to a complex distribution of stress, elevated strains, and cyclical mechanical deformations, affecting its long-term reliability. Despite being a crucial element in all mechanical watch movements, the non-return system appears to have been overlooked in scientific literature, with no studies available on its design, modeling, and micromachining. In this work, we introduce a novel Finite Element Method (FEM) -based design strategy for the ratchet click, systematically refining its geometry and dimensional parameters to minimize peak stress and improve durability. A mechanical simulation model was created to simulate the boundary conditions, contact interactions, and stress distributions on the part. If compared with the standard component, the optimized design exhibits a decrease in peak stress values. The mechanism was micro-machined, and it was experimentally tested to validate the numerical model outputs. The integrated digital–physical approach not only underscores the scientific contribution of coupling advanced simulation with experimental validation of complex micromechanisms but also provides a generalizable method for enhancing performance of micro-mechanical components while preserving their historical design heritage.

## 1. Introduction

Micro components play a crucial role in various industries, including electronics, biomedical engineering, and precision mechanics [1]. These components are frequently fabricated through specialized micromachining processes, which allow for high precision in shaping, structuring, and finishing materials at the microscale. As product miniaturization becomes more prevalent, the demand for reliable manufacturing techniques that achieve accuracy and consistency continues to grow [2].

Micro components can be produced through different technologies, including micro-milling, laser machining, and electrical discharge machining (EDM) [3,4]. Each technique has specific advantages depending on material properties, desired geometries, and production requirements. One of the key aspects in micromachining is the ability to achieve controlled tolerances while minimizing defects such as burr formation and surface irregularities.

The precision of these processes depends on various factors, including cutting parameters, tool geometry, lubrication, and workpiece material properties. Due to the small dimensions of tools and workpieces, micromachining presents many critical issues, such as reduced tool life [5], increased sensitivity to vibrations [6], and difficulties in wear monitoring [7].

Material selection is another critical aspect in micro component manufacturing. Different materials are used according to application needs. Hard-to-cut materials such as titanium alloys, lead-free brass or stainless steels require optimized machining conditions to ensure consistent results and prolonged tool performance. Measurement techniques, such as optical microscopy, are employed to assess surface quality and geometrical and dimensional tolerances [8]. Accurate measurements are essential to evaluate the efficiency of machining processes and to ensure compliance with quality standards. Among the various applications of micromachining, watchmaking represents one of the most demanding fields, requiring extremely high precision and excellent surface finish control [9]. Micro components used in watch mechanisms, such as gears, bridges, and main plates, must adhere to strict dimensional tolerances to guarantee optimal performance. Surface integrity is a key factor, as even minor imperfections can affect performance over time. In addition to geometric accuracy, aspects such as wear resistance, aesthetics, and material compatibility play a significant role in ensuring the durability of watch components [10].

**Figure 1:**
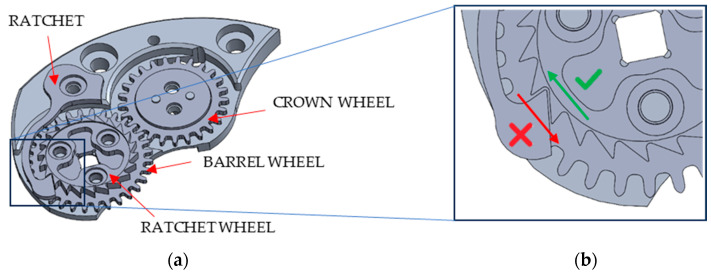
Winding system (**a**) and ratchet wheel teeth shape (**b**).

In a mechanical watch, the winding system stores and regulates energy through a series of interconnected components (Figure 1a). When the crown is turned, motion is transferred through gears and pinions achieving the rotation of the crown wheel. The crown wheel engages with the barrel wheel, transferring the mechanical energy to the main hairspring which stores the elastic potential energy. An anti-reverse mechanism prevents the instantaneous release of the energy of the mainspring, allowing for its gradual discharge. It typically consists of a ratchet click or ratchet, constrained to the movement structure, and a ratchet wheel coaxial with the barrel and joined on it. The ratchet wheel engages with the ratchet avoiding the rotation in anticlockwise direction thanks to the particular shape of its teeth (Figure 1b). The ratchet engages with the ratchet wheel to secure the winding system, making it a critical component exposed to fatigue stress. During charging, the ratchet undergoes elastic deformations, and their entity depends on its shape and on the shape of the ratchet wheel teeth. To prevent plastic deformation and irreversible failure, it is essential that the ratchet remains within its elastic range, avoiding excessive strain that could compromise performance. This study aims to develop a ratchet mechanism used in winding systems by investigating geometric configurations that reduce stress concentrations and improve overall performance. The challenge lies in modifying the geometry of the ratchet and ratchet wheel in order to identify configurations that minimize stress concentrations and ensure the proper functioning of the winding system. Finite Element Method (FEM) simulations can be employed to evaluate different designs and optimize structural integrity. In the literature, several studies regarding the usage of numerical methods for solving complex engineering problems are available. Argyris (1954) and Turner et al. (1956) were the first to publish the use of such techniques for the aircraft industry [11]. Subsequently, the FEM has been extended to other areas of solid mechanics, and it has become a consolidate method for design optimization in many engineering and applied science fields [12].

FEM simulations are widely employed in the study of gear dynamics. For example, Ma et al. (2015) analyzed multiple strategies for predicting crack propagation in gear teeth by combining analytical models with FEM-based approaches [13]. The reviewed studies include simulations on spur, helical, and spiral bevel gears, with crack propagation assessed using both 2D and 3D models. Notably, the FEM analyses incorporated elastoplastic fracture mechanics, going beyond linear elastic assumptions to capture more realistic crack growth behavior under operational loading conditions. Another study was conducted by Liu et al. [14] on gear transmission systems using FEM simulations. In this case as well, the researchers used FEM models to evaluate the effects of tooth root cracks. Also, Ooi et al. applied the FEM to analyze different gear train configurations used in portal axle systems, with the objective of investigating their modal behavior, gear tooth bending stress, and contact stress [15]. Through modal and static stress analyses, the researchers measured natural frequencies, mode shapes, and stress distributions under varying angular positions and contact conditions, demonstrating that the FEM results aligned closely with classical analytical model, confirming the reliability of the simulations. However, the above mentioned studies focus on larger-scale gears and do not address applications in the horology field, where micro gear components are typically employed. According to the German standard VDI 2731 [16], micro gears are defined as gears, which have almost two of the three following characteristics: external dimensions (e.g., diameter or edge length) < 20mm, module < 200 μm or structural details < 100 μm. Haefner et al. [17] used FEM simulations on micro-gears to develop a method for predicting their service life, combining characteristic loads obtained from FEM models with experimental test data. Furthermore, only few studies regard the application of numerical simulations to the functioning of anti-reverse mechanisms [18]. Piovesan and Alladi [19] performed a Finite Element Analysis of a ratchet used in a passive ankle exoskeleton designed to reduce metabolic cost during walking. The FEM study focused on critical load conditions during gait and revealed that high contact stresses—especially on the pawl and ratchet face—could exceed the material yield strength (AISI 1020 steel), highlighting the need for careful material selection and mechanical design refinement. However, the ratchet mechanism was implemented at a mesoscopic scale and within a complex biomechanical system, with limited focus on structural miniaturization or wear resistance. Xu R. et al. [20] studied a curved beam snap-fit structure, similar to a ratchet click mechanism, using Finite Element Method (FEM) simulations. They employed FEM to investigate the mechanical behavior of rotational snap-fit mechanical metamaterials (RSMMs), aiming to characterize their torque-angle response, stress distribution, and multi-stability under torsional loading. The results demonstrated a high level of agreement between simulation, theoretical analysis, and experimental observations. Nevertheless, the use of polymeric materials and the geometrical complexity of the structure may limit its applicability in precision micro-mechanics or in load-bearing applications involving metallic components.

Other recent works, such as Qi et al. [21] and Kahraman and Küçük [22], have applied topological optimization techniques to macro-scale ratchet systems. These studies, however, do not address the scale-dependent mechanical and material issues relevant to micro-fabricated metallic devices.

These contributions provide valuable insight into ratchet-based mechanisms across various applications and scales. While such mechanisms have been extensively studied at the macro- and meso-scale, and often involve polymeric systems, the literature lacks detailed numerical analyses and mechanical design approaches specifically focused on metallic micro-scale ratchet mechanisms—particularly those employing stainless steel.

**Figure 2:**
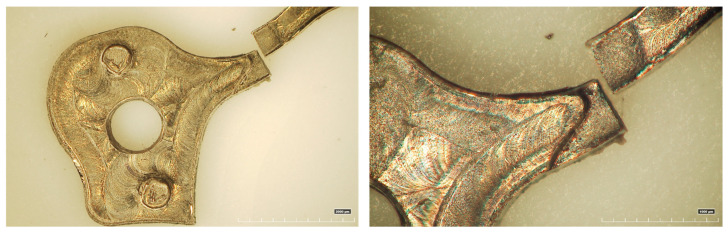
Breaking of the original ratchet.

This work aims to fill that gap by proposing a miniaturized anti-reverse device based on a pawl-ratchet principle, tailored for micro-mechanical applications that demand high precision, compactness, and durability. A FEM model was developed using Deform 3D V14.0 to study the stresses and deformations acting on a specific anti-reverse mechanism of a watch movement, the one depicted in Figure 1. The study case was selected considering some criticisms about the functioning, including a breakage of the stainless steel ratchet during some tests, shown in Figure 2. Regardless of the specific geometry of the case study, the aim of the paper is to share a geometric optimization approach for micro-scale mechanical systems, based on large-deformation FEM modeling and robust statistical methods. To the best of the knowledge of the authors, this study constitutes the first documented publication addressing the non-return mechanism in mechanical watch movements. The numerical approach made it possible to optimize the geometry and dimensions of the ratchet and the wheel both in a FE environment, before micro-machining the solution. Different geometries were simulated to identify the optimal configuration. Once the best design was identified, the anti-reverse mechanism was micro-machined and tested, ensuring a correct functioning during the time without investing a long period for experimental tests.

## 2. Materials and Methods

The initial configuration, visible in Figure 1, of the original anti-reverse mechanism was modeled by using the commercial software DEFORM 3D V14.0. This step is essential to evaluate the key factors influencing the stresses and deformations of the ratchet and the ratchet wheel. A static mechanical simulation was implemented by considering the wheel and the ratchet as elasto-plastic bodies. They were both manufactured in AISI301 stainless steel (annealed sheet with a thickness of 1.2 mm, R_p0.2_ = 500 MPa; R_m_ = 750 MPa declared by the supplier datasheet), material available in the standard software library. The plastic behavior of the material included strain and strain rate hardening effects both. Table 1 summarizes the key geometrical features of the components and the overall dimensions *L* and *W_h_* of the ratchet.

**Figure 3:**
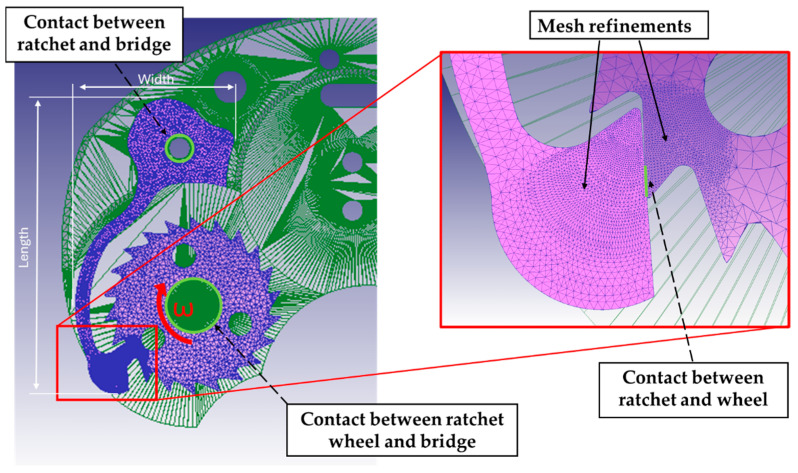
FEM model of the initial configuration of the anti-reverse mechanism and a detail of the mesh refinements.

In regard to the boundary conditions, to simplify the configuration shown in Figure 1, both components were mechanically constrained to the structure of the watch movement, modeled as a rigid body: the barrel wheel was not considered, and the ratchet wheel was directly attached to a coaxial pin on the bridge (i.e., a part of the structure of a watch movement with the main plate). The ratchet was fixed to the structure inside a profiled pocket on the bridge. Figure 3 shows the configuration adopted for numerical modeling. Since the ratchet wheel has *z* teeth, a clockwise wheel rotation of 18° with a speed ω = 10 Rotations Per Minute (RPM) was modeled, to simulate a complete cycle of deformation of the ratchet. In particular, since a single tooth engages with the ratchet for a rotation equivalent to 16.4° (which is 360°: 22 teeth), the simulation of a rotation equal to 18° allowed to simulate also the descendent phase to complete the cycle of deformation of the ratchet. No external loads were applied, since the deformation of the ratchet was promoted directly by the contact with the wheel. The torque applied by the barrel wheel was not considered since its effect on the mechanical behavior of the system during the charging phase is negligible. Both the ratchet and wheel were meshed by using 100k tetrahedral elements. The distribution of the element size was improved by using the mesh windows: a region with a high density of mesh elements was localized on the head of the ratchet (with a maximum size ratio equal to 10); similarly, a local mesh refinement was also adopted on the tooth of the ratchet wheel (with a maximum size ratio equal to 100). The minimum element size for both components was 10 μm. Friction between the components was modeled by using the Coulomb model, adopting a coefficient of 0.7 for the friction between the ratchet and the wheel and a value of 0.5 for the contact between both components and the bridge, made from lead-free brass [23].

The simulation of the initial configuration of the original anti-reverse mechanism allowed to detect some criticalities related to the geometry of the micro-components, exploited in the Section 3. A critical revision of the design was performed achieving a pre-optimization of the ratchet and of the wheel. Once the components were re-designed considering the mentioned optimizations, a numerical analysis was performed to identify the best value of two key geometrical parameters of the wheel: the height *h* and the fillet radius *r* of the teeth (see Figure 3). The height *h* determines the amount of deflection of the ratchet, since higher *h* requires higher deflection to allow the ratchet to overcome the tooth of the wheel. The radius *r* influences the severity of the transition between the ascent and descent phases of the ratchet, and, in particular, higher *r* mitigates stress concentration and ensures a smoother transition. For both parameters, two different values were considered, as reported in Table 2.

The values were chosen by decreasing *h* and increasing *r* if considering the original component (Table 1). For the fillet radius r, the original value (0.02 mm) was first doubled to 0.04 mm. An additional doubling to 0.08 mm was initially planned, but this was later adjusted to 0.07 mm to preserve the aesthetic integrity of the design.

Regarding the tooth height h, the values of 0.26 mm and 0.30 mm were determined based on the results of the initial numerical simulation performed on the original system. These values correspond to the configuration observed at the instant of plastic deformation of the ratchet, providing a relevant reference frame for the improvement analysis. Four FEM simulations were performed to optimize the ratchet mechanism, following a 2^2^ complete factorial plan described in Table 3. To perform the comparative analysis on the effect of the wheel geometry, the model previously described was simplified by considering the wheel as a rigid body and the ratchet as an elastic one. The other setting of the model was kept constant, as in the initial elastoplastic simulation. Finally, the best configuration was modeled by considering the elastoplastic behavior of both components, as was done with the initial configuration, to validate the Design of Experiments DOE approach.

Once the numerical optimization was carried out, the physical components with the final design were micro-machined through micro-milling operations. The ratchet and ratchet wheel were manufactured using a 5-axis machining center (KERN Pyramid Nano, Eschenlohe, Germany) equipped with a CNC controller (HEIDENHAIN iTNC 530, Traunreut, Germany). This ultra-precise machine operates at spindle speeds of up to 50,000 rpm with a maximum torque of 1.5 Nm, making it suitable for high-precision micro-milling with small-diameter tools. The axis movement system is based on hydrostatic guideways, providing a positioning accuracy of 2 µm. The pyramid-shaped structure ensures high machine rigidity, reducing vibration during machining—an essential condition for micro-tool integrity [24]. The machining environment was maintained at a controlled temperature of 20 ± 0.5 °C and 35% relative humidity. Thermal stability during operations was further ensured by a temperature management system with five independent cooling circuits.

Both components of the non-return system were manufactured using AISI 304 stainless steel. All tools (micromills and microdrills) were mounted on tool holders (Big Kaiser HSK-F63-MEGA6N-90, Rümlang, Switzerland), ensuring a maximum run-out of 3 µm with overhangs up to four times the tool diameter. The micro end mills used were supplied by the manufacturer Tecnica Srl (Brescia, Italy), while the drilling tools were provided by Mikron (Agno, Switzerland). The ratchet was machined using a 0.88 mm diameter drill and a 0.80 mm diameter end mill. The ratchet wheel was produced using a 1.10 mm diameter drill, a 0.80 mm end mill, and a 0.20 mm end mill. Cutting parameters for each tool were chosen based on the manufacturer’s recommendations. Table 4 describes the characteristics of the tools. About the parameters, cutting speed, feed rate, and depth of cut were modified if compared to the setup employed to machine the original components of the anti-reverse mechanism in order to comply with the paradigm of High Speed Machining (HSM): feed rates were increased, reducing the depth of the cut. Table 5 lists the original and the new set of process parameters.

CNC programming was carried out with Solidworks 2024 CAM © (Dassault Systèmes, Waltham, MA, USA). Dimensional verification of the manufactured components was performed using a Mitutoyo Quickscope CMMO (Kawasaki, Japan) coordinate measuring machine. The profiles of the fabricated components were captured and compared with the corresponding CAD models. This comparison supported an evaluation of the micromachining accuracy. The acquired profiles were matched onto the CAD geometries, and deviations were measured based on a limited set of points distributed along the profiles. Specifically, the average offset and its standard deviation were computed.

## 3. Results

**Figure 4:**
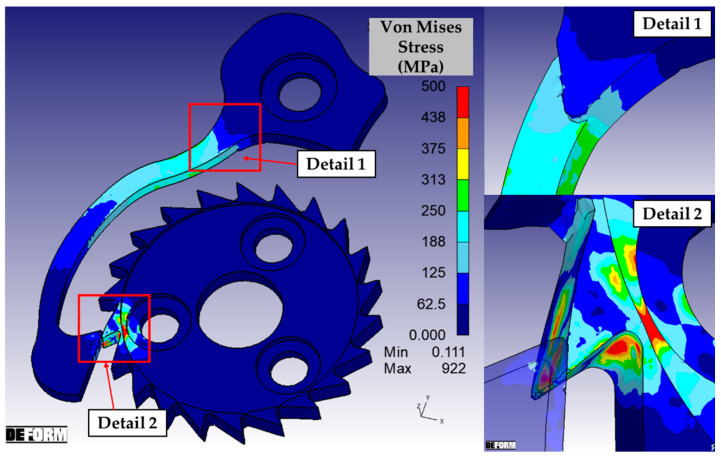
Results of the simulation of first initial configuration, with a detailed view of the discontinuity on the ratchet where there is an abrupt variation in the section and a magnification on the tooth wheel.

Figure 4 shows the results of the simulation of the original anti-reverse mechanism. The most heavily loaded region results located on the tooth of the ratchet wheel, with wide portions subjected to stresses exceeding the yield strength of the material. In particular, the inner region at the base of the tooth experiences severe bending, while the outermost area, in contact with the ratchet, undergoes significant compressive deformation. The stress on the terminal portion of the ratchet is significantly lower, but on the other hand the shank is interested by non-neglectable bending effect. It was possible to identify a specific area on the ratchet where the highest stress occurs. In particular, the greatest stresses were localized on the part of the ratchet where there is an abrupt variation in the section. This geometrical discontinuity modifies the flow of the stress, generating a typical behavior of solid mechanics known as stress concentration [25]. This phenomenon is linked to geometric discontinuities, such as internal corners, holes, and sudden changes in the cross-sectional area of the components, as well as unintentional damage like nicks, scratches, and cracks [26].

**Figure 5:**
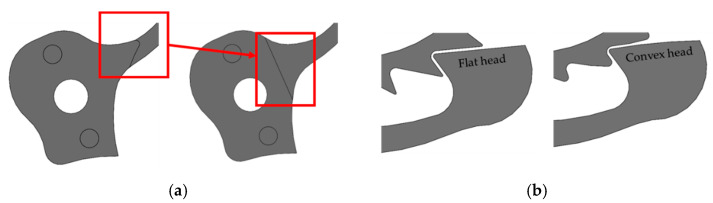
Shoulder geometry before and after the optimization (**a**); ratchet head and teeth geometry before and after the optimization (**b**).

These considerations led to a modification of the ratchet geometry, relocating the shoulder to a section of the ratchet with a larger cross-sectional area, as visible in Figure 5a, shifting the stress raiser effect in a less critical zone of the ratchet. In addition to this improvement, a pre-optimization of the ratchet head geometry was performed. The surfaces of the ratchet and ratchet wheel in contact during the sliding were modified, changing from flat to slightly convex surfaces (see Figure 5b). In fact, a flat contact surface on the ratchet head tends to generate highly localized contact stresses, especially near the edges of the contact area, which can lead to plastic deformation, surface fatigue, and premature failure. As demonstrated by Brezeanu [27] through FEA, introducing slight curvature to the contact surface can significantly reduce stress concentrations by distributing the load more evenly across the interface. In this way, concentrated contact stresses are minimized, which increases the component lifespan and reduces sliding wear.

**Figure 6:**
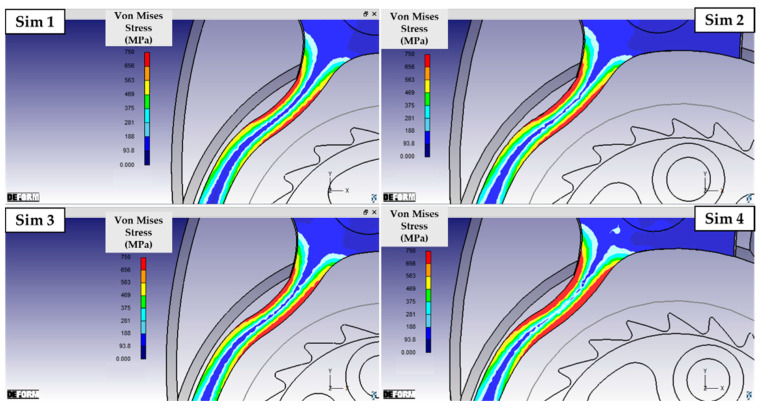
Von Mises stress distribution in the portion of the ratchet with maximum bending.

**Figure 7:**
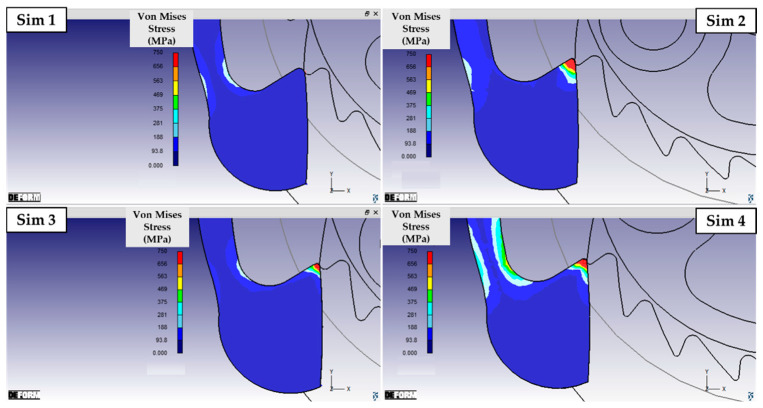
Von Mises stress distribution in the end region of the ratchet in contact with the wheel teeth.

**Figure 8:**
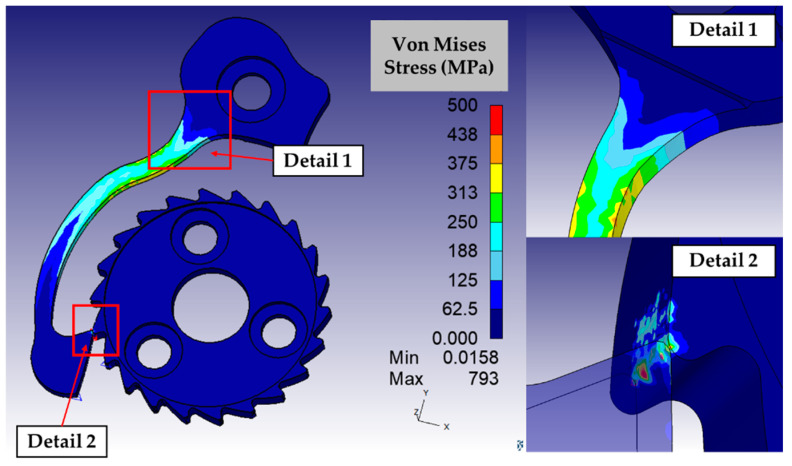
Results of the simulation of final configuration of the anti-reverse mechanism, with a detailed view of the discontinuity on the ratchet and a magnification on the tooth wheel.

As described in Section 2, the pre-optimized geometries were used to perform a DOE 2^2^ analysis varying the height *h* and the fillet radius *r* of the wheel teeth. Figure 6 and Figure 7 show the results of the equivalent deformation of the ratchet in the four simulations summarized in Table 3. The figures show the von Mises stress using a custom scale limited to 750 MPa, corresponding to the ultimate strength of the material. Modeling the wheel as a rigid body and the ratchet as an elastic component allowed a reduction in computation time without affecting the comparative analysis. As hypothesized, the geometry and size of the wheel tooth is crucial, as they directly affect how much the ratchet deforms during wheel rotation. Figure 6 in particular illustrates the stress distribution in the portion of the ratchet interested by the maximum bending. By comparing the simulations with the same tooth fillet radius r but different heights h of the tooth (Sim 1 versus Sim 3, and Sim 2 versus Sim 4), it can be observed that increasing h results in greater bending deflection. Consequently, the localized stresses on the shank of the ratchet increase, expanding the region approaching failure both across the section and along the profile of the ratchet. A very similar effect can be observed when considering teeth with the same height h but lower fillet radii r (Sim 1 versus Sim 2, and Sim 3 versus Sim 4): a sharper tooth forces the ratchet to undergo a more abrupt deformation, resulting in higher stress. Therefore, when considering the deformation of the ratchet shank—where the original component experienced damage—the solution tested in Simulation 1 proved to be the most effective configuration (minimum h and maximum r). Figure 7 confirms these observations: it is evident that the stress in the end region of the ratchet is significantly lower in Simulation 1 compared to all other cases. In particular, this is the only configuration in which failure due to compression of the ratchet tip is not predicted. Relative to this condition, both the increase in h (Sim 2) and, especially, the reduction in r (Sim 3) lead to higher local stress at the tip, compromising its integrity. The combined effect of both modifications (Sim 4) not only causes damage at the extremity but also substantially increases the bending deflection of the final portion of the ratchet shank, where the head develops. In conclusion, the geometry of tooth n° 1 is the most advantageous configuration from all perspectives, and it was employed to perform the final elasto-plastic simulation, shown in Figure 8.

The elasto-plastic simulation confirmed the improvements determined by the design optimization. The most evident advantage is related to the solicitation on the tooth of the wheel: if compared with the original configuration, visible in the detail 2 of Figure 4, the reduction in the stress on the tooth is high. The maximum solicitation on the shank of the ratchet is slightly higher than the original configuration (detail 1 in Figure 4), but it resulted in a lower strength than the yield strength limit. To correctly interpret this data, it is also necessary to consider that the original model is affected by a plastic deformation of the tooth, which reduces the bending of the ratchet as a consequence. Detail 1 of Figure 8 shows how the shifting of the variation in the section of the ratchet ensures avoiding the stress concentration effect. The FE-driven design activities of the anti-reverse micro mechanism were considered completed and the components were consequently micro manufactured.

**Figure 9:**
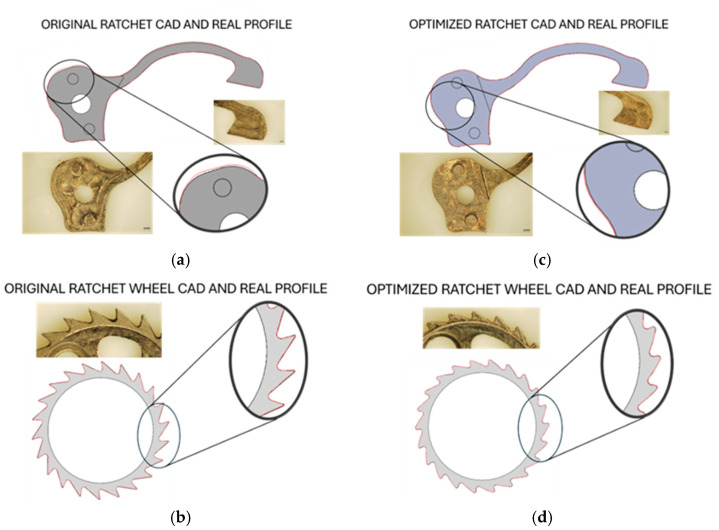
Ratchet profile before (**a**) and after optimization (**c**); ratchet wheel profile before (**b**) and after optimization (**d**).

Figure 9 shows the micro-machined ratchet and ratchet wheel—both in their original (Figure 9a,b) and optimized configurations (Figure 9c,d). The CAD profiles were overlapped with the actual profiles of the real components, which are highlighted by red lines. The offset was measured by considering 22 points for the wheels (one for each tooth) and 32 points for the ratchet, equally distributed along the profiles. The results are listed in Table 6, expressed as the average values and the standard deviation of the measurements. Negative offset means that the actual components resulted in undersizing compared with the CAD geometry. The average offset between CAD and the actual profile ranged between −0.010 mm and 0.027 mm; considering the tool run-out effect (i.e., the deviation from the nominal trajectories by the actual trajectories of the tool during the rotation), the error on the diameter of the micro-mill and the error related to the measurement, the offset is acceptable and it does not affect the functionality of the anti-reverse mechanism.

The optimized components were machined by changing the strategy, choosing high feed and low depth of cut. As a result, the offset decreased for both the ratchet and the wheel. The deviation for the optimized components ranged between 0.007 and 0.012 mm, with a considerable improvement compared to the original components. To assess the reliability of the proposed mechanism, an experimental test campaign was carried out. Forty watches were assembled using the mechanism and operated continuously for a minimum of two weeks. Each unit underwent roughly 400 h of functioning, corresponding to about 1800 cycles of mechanical deformation. No structural failures or operational malfunctions were recorded. These results demonstrate that the mechanism performs consistently under extended usage conditions.

## 4. Conclusions

This study presented a FEM-driven optimization approach for the improvement of a micromachined ratchet click mechanism employed in mechanical watch movements. Starting with the analysis of the original configuration, which exhibited stress concentrations and potential points of mechanical failure, numerical simulations were used to identify geometric improvements both in the ratchet and in the ratchet wheel. The integration of design modifications—such as the repositioning of the section transition, the use of convex contact surfaces, and the optimization of wheel tooth height and radius—resulted in significant reductions in peak stresses and improved load distribution.

The implementation of a full-factorial DOE enabled the identification of the most advantageous configuration, which was then validated through elasto-plastic simulations. The subsequent micro-milling of the optimized components, performed under HSM conditions, demonstrated the feasibility of the optimized design. Dimensional measurements confirmed the fidelity of the manufacturing process to the CAD models, with significantly reduced deviations compared to the original components.

Overall, the proposed methodology confirms the effectiveness of using large-deformation FEM simulations and micromachining process optimization to enhance the performance and reliability of precision components, even in heritage-based systems such as mechanical watches. The approach can be readily extended to other micro-mechanical assemblies where stress concentrations and dimensional constraints critically affect the functionality and lifespan of the system.

The results confirm that the proposed ratchet mechanism performs reliably under repeated mechanical loading. Future work will focus on designing alternative winding systems with greater efficiency and broader applicability. To improve the durability assessment, the experimental procedure could be enhanced through automated actuation setups capable of running high-cycle fatigue tests over extended timeframes. In the current study, numerical simulations were validated through direct experimentation: the mechanism was implemented in forty wristwatches and tested over more than 400 h of operation and 1800 deformation cycles, with no failures observed. In subsequent research, analytical models could be introduced to complement experimental data and provide deeper insight into stress distribution and material behavior.

## Figures and Tables

**Table 1:** Dimension of the components of the original anti-revere mechanism.

Ratchet Wheel		Ratchet	
Thickness *t_w_* (mm)	0.60	Thickness *t_r_* (mm)	0.30
External Diameter *D_e_* (mm)	5.88	Wideness *w* (mm)	0.45
Number of teeth *z*	22	Length *L* (mm)	9.78
Height of the teeth *h* (mm)	0.40	Width *W_h_* (mm)	5.12
Fillet radius of the teeth *r* (mm)	0.02		

**Table 2:** Values of *h* and *r* employed in the numerical analysis.

Parameter	Level 1	Level 2
Height of the teeth *h* (mm)	0.30	0.26
Fillet radius of the teeth *r* (mm)	0.04	0.07

**Table 3:** Summarization of the simulations and the related *h* and *r*.

Simulation Test	*h* (mm)	*r* (mm)
1	0.26	0.07
2	0.26	0.04
3	0.30	0.07
4	0.30	0.04

**Table 4:** List of the tools employed during micromachining of the components.

Tool	Diameter (mm)	Manufacturer	Code	Material	Coating
Mill	0.20	Tecnica	FCPRC0.2G4L52Z2	WC	AlTiSiN
Mill	0.80	Tecnica	FCPRR0.8G6L52Z2	WC	AlTiSiN
Drill	0.88	Mikron	2.MD.210088.1	WC	Multilayer PVD
Drill	1.10	Mikron	2.MD.200110.1	WC	Multilayer PVD

**Table 5:** List of the process parameters.

Tool	Diameter (mm)	Vc (m/min)	fz (mm/z*rev)	ap (mm)	Vc (m/min)	fz (mm/z*rev)	ap (mm)
		*Original components*			*Optimized components*		
Mill	0.20	25	0.001	0.02	20	0.004	0.01
Mill	0.80	60	0.005	0.1	40	0.01	0.03
Drill	0.88	60	0.016	0.1	40	0.016	0.1
Drill	1.10	60	0.023	0.1	40	0.023	0.1

**Table 6:** Average values and the standard deviation of the measurements.

Component	Average (mm)	Standard Deviation (mm)
Original Ratchet	0.027	0.017
Optimized Ratchet	0.012	0.005
Original Ratchet Wheel	−0.010	0.004
Optimized Ratchet Wheel	0.007	0.003

## Data Availability

The data presented in this study are available on request from the corresponding author due to copyright reasons.
